# De Novo aneurysms in long–term follow-up CT-angiography of patients with clipped intracranial aneurysms

**Published:** 2012-11

**Authors:** Alireza Zali, Reza Jalili Khoshnood, Afsaneh Zarghi

**Affiliations:** ^*a*^Department of Neurosurgery, Functional Neurosurgery Research Center, Shahid Behesti University Of Medical Sciences, Tehran, Iran.

## Abstract

**Background:**

This study was conducted to evaluate the de Novo aneurysm formation in the long-term follow-up of patients with clipped intracranial aneurysms.

**Methods:**

A total of 459 patients underwent the clipping of ruptured cerebral aneurysms in our institution during 1997 -2008. Of them, 119 patients were available in good condition and agreed to undergo 64 detector row computed tomography (CT) angiography. In addition, 8 patients with subarachnoid hemorrhage (SAH) underwent CT angiography.

**Results:**

The mean ± SD interval from surgery was 7.2±2.3 years for CT-angiography controlled patents. De novo aneurysms were detected in 5 of 119 (4.5%) patients and 4 of 8 patients with new subarachnoid hemorrhage. A history of multiple aneurysms was associated with the de novo aneurysm formation (p less than 0.001).

**Conclusions:**

The risk of the de novo aneurysm formation in patients with clipped aneurysm is significant in long term follow-up. CT-angiography can be used as a non-invasive method for detection of de novo aneurysms in these patients.

**Keywords:**

Cerebral aneurysm, Ct-angiography, De Novo aneurysm, Surgical clipping


					Volume 4, Suppl. 1 Nov 2012
Publisher: Kermanshah University of Medical Sciences
URL: http://www.jivresearch.org
Copyright:
Congress of Iranian Neurosurgeons, 14-16 November 2012, Kermanshah, Iran
Paper No. 53
De Novo aneurysms in long-term follow-up CT-angiography
of patients with clipped intracranial aneurysms
Alireza Zali a,*
, Reza Jalili Khoshnood a
, Afsaneh Zarghi a
a
Department of Neurosurgery, Functional Neurosurgery Research Center, Shahid Behesti University Of Medical Sciences,
Tehran, Iran.
Abstract:
Background: This study was conducted to evaluate the de Novo aneurysm formation in the long-term follow-up of
patients with clipped intracranial aneurysms.
Methods: A total of 459 patients underwent the clipping of ruptured cerebral aneurysms in our institution during
1997 -2008. Of them, 119 patients were available in good condition and agreed to undergo 64 detector row
computed tomography (CT) angiography. In addition, 8 patients with subarachnoid hemorrhage (SAH) underwent CT
angiography.
Results: The mean  SD interval from surgery was 7.22.3 years for CT-angiography controlled patents.
De novo aneurysms were detected in 5 of 119 (4.5%) patients and 4 of 8 patients with new subarachnoid
hemorrhage. A history of multiple aneurysms was associated with the de novo aneurysm formation (p less than
0.001).
Conclusions: The risk of the de novo aneurysm formation in patients with clipped aneurysm is significant in long term
follow-up. CT-angiography can be used as a non-invasive method for detection of de novo aneurysms in these
patients.
Keywords:
Cerebral aneurysm, Ct-angiography, De Novo aneurysm, Surgical clipping
* Corresponding Author at:
Alireza Zali: Functional Neurosurgery Research Center, Shohada Hospital, Tajrish Avenue, Tehran. Iran, Email: FNRC.AZ@hotmail.com, (Zali A.).

				

